# Emergence of rigidity percolation in flowing granular systems

**DOI:** 10.1126/sciadv.adh5586

**Published:** 2023-09-01

**Authors:** Hor Dashti, Abbas Ali Saberi, S. H. E. Rahbari, Jürgen Kurths

**Affiliations:** ^1^ Australian Institute of Bioengineering and Nanotechnology, The University of Queensland, Brisbane, QLD 4072, Australia.; ^2^ School of Physics, Korea Institute for Advanced Study, Seoul 02455, Korea.; ^3^ Department of Physics, University of Tehran, P. O. Box, 14395-547 Tehran, Iran.; ^4^ Max Planck Institute for the Physics of Complex Systems, 01187 Dresden, Germany.; ^5^ Potsdam Institute for Climate Impact Research, Potsdam, Germany.; ^6^ Department of Physics, Humboldt University, Berlin, Germany.

## Abstract

Jammed granular media and glasses exhibit spatial long-range correlations as a result of mechanical equilibrium. However, the existence of such correlations in the flowing matter, where the mechanical equilibrium is unattainable, has remained elusive. Here, we investigate this problem in the context of the percolation of interparticle forces in flowing granular media. We find that the flow rate introduces an effective long-range correlation, which plays the role of a relevant perturbation giving rise to a spectrum of varying exponents on a critical line as a function of the flow rate. Our numerical simulations along with analytical arguments predict a crossover flow rate ${\dot{\gamma }}_{c}≃10^{-5}$ below which the effect of induced disorder is weak and the universality of the force chain structure is shown to be given by the standard rigidity percolation. We also find a power-law behavior for the critical exponents with the flow rate $\dot{\gamma }>{\dot{\gamma }}_{c}$.

## Teaser

Flow rate induces long-range correlations to shape the force chain structure in the jammed granular media.

## INTRODUCTION

One of the principal characteristics of particulate matter, such as granular media and emulsions, is the loss of energy upon every collision, which makes particulate matter an intrinsically nonequilibrium system. However, amorphous particulate materials exhibit many equilibrium-like features, such as the long-range spatial correlations, a ubiquitous property of equilibrium systems at their critical point. These correlations have been mainly discussed in the context of (i) interparticle forces and (ii) stress components. However, understanding how these long-range correlations influence static and dynamic properties of amorphous materials is in its infancy; nonetheless, it promises a fascinating avenue of research. This is a subject of fundamental interest for solid mechanics and rheology (*1*), with applications in resource industries (*2*), seismicity (*3*), material processing, tissue mechanics (*4*), health care, soft robotics, and topological metamaterials (*5*).

In a dense flow regime, unlike its dilute counterpart, grains experience multiple and enduring contacts at all times without the possibility of free flights during an induced shear deformation. This leads the kinematic field of dense granular flows to exhibit complex spatiotemporal correlations and spontaneous formation of quasi-rigid clusters in the flow (*6*, *7*). The key to understanding the origins of such correlations is the characterization of interparticle force networks as the backbone of the stability and rigidity of amorphous materials. Percolation theory (*8*), which describes the connectivity behavior of a network when nodes or links are added, has been widely used for this purpose.

In a seminal work, using photoelastic disks, Majmudar and Behringer (*9*) found long-range spatial interparticle force correlations for systems subjected to pure shearing and short-range correlations for systems under isotropic compression. This work has been ensued by many experimental and numerical studies using percolation theory for the network of interparticle forces, in which a bond is attributed between two adjacent particles if their interparticle force exceeds a threshold, i.e., *f* ≥ *f*_t_. These studies can be divided into two main categories: (i) Ostojic *et al.* (*10*) found long-range correlations of interparticle forces; Kovalcinova *et al.* (*11*) also found long-range correlations but with critical exponents not consistent with the previous study in (*10*). Tong *et al.* (*12*) investigated percolation of background forces contributing to mechanical equilibrium in a glass model and found scaling exponents other than the random percolation universality class. In stark contrast to these studies, in category (ii), Pathak *et al.* (*13*) performed a similar numerical analysis on isotropically compressed spheres in two and three dimensions and found short range correlations consistent with the random percolation universality class. Accordingly, a long-range correlation with debated exponents is found in category (i), and a short-range correlation consistent with the standard random percolation universality class is reported in category (ii).

There is a large body of studies devoted for percolation of contacts between particles (*14*, *15*); the contact network is the asymptotic limit of the interparticle force network when *f*_t_ → 0. For instance, Shen *et al.* (*16*) found that 2*d* systems under isotropic compression undergo a connectivity percolation at a density ϕ_P_ ≪ ϕ_J_ with a correlation exponent different from that of random percolation. More recent studies have applied techniques of complex networks, such as centrality measures, to investigate the interplay between various types of centralities and local elastic properties (*17*).

Whereas the connectivity percolation examines the possibility of a spanning cluster, a more stringent condition is required for rigidity percolation (RP): The spanning cluster must be mechanically rigid. The RP was originally proposed to describe the emergence of solidity of covalent network glasses (*18*). In athermal systems, rigidity is commonly explored by Hessian, in which the absence of system-spanning zero-cost modes infers rigidity (*19*). Recently, the RP has found many modern applications in phase transitions associated with rigidity in, as diverse systems as, mechanical topological metamaterials (*5*), protein folding (*20*), jamming by compression (*21*), gelation via attractive interactions (*22*), and a generalized RP for frictional particles (*23*, *24*). The universality class of RP is determined by a graph theoretic technique known as the pebble game (*18*). RP is a second-ordered phase transition for the bond-diluted generic triangular lattice, and the exponents ν_RP_ = 1.21 ± 0.06 and β_RP_ = 0.18 ± 0.02 can be obtained by using the cluster moment definitions (*18*). Providing the correctness of the hyperscaling relations γ = *d*ν − 2β and η = 2(1 + β/ν) − *d* (*25*), one can calculate the two other important exponents γ_RP_ = 2.06 ± 0.08 and η_RP_ = 0.30 ± 0.02. To the best of our knowledge, scaling properties given by the standard RP have never been retrieved in off-lattice simulations of frictionless spheres. Moreover, many studies have investigated a possible connection between jamming in sphere packings and RP; however, it is shown that RP and jamming are distinct (*21*).

Here, we report that, at the low limit of the flow (shear) rate below a certain crossover value ${\dot{\gamma }}_{c}$, the scaling properties of the percolation network of interparticle forces in particulate matter comply with those of standard RP universality class. We argue that the flow rate acts as a relevant perturbation, resulting in a spectrum of varying exponents on a critical line. These results shed light on the controversy of the nature of percolation in isotropically compressed packings, because the compression rate may similarly act as a relevant perturbation, and this would explain the range of different exponents reported for the percolation transition in isotropically jammed packings (*10*–*12*, *14*, *15*). Above ${\dot{\gamma }}_{c}$, the induced disorder by the flow rate is long range enough to drive the universality class of the system with continuously varying critical exponents with $\dot{\gamma }$. However, the critical exponents, including the one describing force-force correlation and the fractal dimension of the spanning force cluster, remain unaffected in the whole range of the considered flow rates. Our results should pave the way to improve elastoplastic models, vastly used in material science and engineering, to include the interplay between the flow rate and force-force correlations as ingredients of the elastoplastic models. Our study can be suitably extended for the understanding of the transitions between different inherent structures and constraint networks in disordered solids (*26*) when the flow rate is considered as the control parameter. This establishes further analogies between glass and granular physics and their response to external deformations.

## RESULTS

**Fig. 1.**
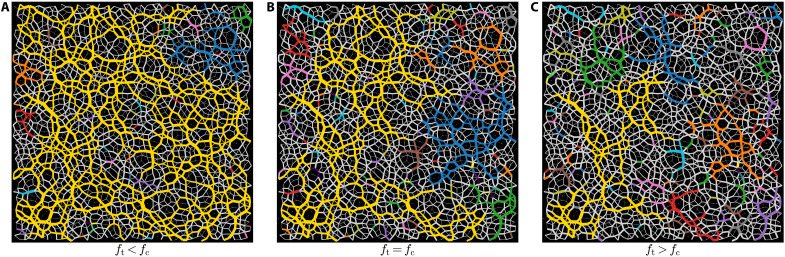
Snapshots of network of interparticle forces. Each particle is regarded as a node, and the corresponding interparticle force must be larger than a threshold force, *f* > *f*_t_, to grant a link. By increasing the threshold force, *f*_t_, weak interactions are diluted, and the largest cluster in the system undergoes a percolation transition at *f*_t_ = *f*_c_. We identify clusters using a union-find clustering algorithm (*60*), adopted for off-lattice simulations. In these snapshots, the width of a link is proportional to the strength of the interparticle force. The color coding is performed according to the following rules: Diluted weak interactions, for *f* < *f*_t_, are depicted by gray color, the largest cluster is yellow, and the rest of clusters are marked by various other colors. Three different snapshots are shown for (**A**) *f*_t_ < *f*_c_, (**B**) the network is shown at the onset of the percolation transition *f*_t_ = *f*_c_, and (**C**) *f*_t_ > *f*_c_. In these snapshots, the number of particles is *N* = 2048; the packing fraction is ϕ = 0.86 and $\dot{\gamma }=10^{-6}$; the threshold force in (A) to (C) is *f*_t_ = 0.00976, 0.01084, and 0.01193, respectively.

We perform molecular dynamics simulations of two-dimensional athermal frictionless bidisperse disks in a simple shear flow using the Large-scale Atomic/Molecular Massively Parallel Simulator (LAMMPS) (*27*). Details on the simulations are given in Methods. We build a network of interparticle forces by diluting weak interactions: Each particle is considered as a node, and a contact is regarded as a link if the interparticle force *f* exceeds a putative threshold, i.e., *f* ≥ *f*_t_. In a jammed state when *f*_t_ is small, this network connects most of the particles. By increasing *f*_t_, as a result of bond dilution, the largest cluster undergoes a percolation transition at *f*_t_ = *f*_c_. In Fig. 1 (A to C), we display three snapshots for *f*_t_ < *f*_c_, *f*_t_ = *f*_c_, and *f*_t_ > *f*_c_, respectively. The width of each link is proportional to the magnitude of the interparticle force. The gray color corresponds to diluted weak forces, *f* < *f*_t_. Non-gray colors correspond to existing bonds where *f* ≥ *f*_t_. The largest cluster is marked by the yellow color. In Fig. 1B, the largest cluster at the percolation transition contains most of strong forces in the system. One can see that the network consists of various types of polygons; triangles seem to have the largest population, in accordance with (*15*). In some cases, these triangles act as hinges connecting neighboring polygons. When the threshold is large (Fig. 1C), most of the polygons in the largest cluster are washed out, and chiefly linear structures remain. This emphasizes that the force network consists of two subnetworks: filamentary linear structures carrying most of the stress and polygonal structures playing the role of stabilizer and hinges for the strong filamentary chains. This is consistent with previous studies (*15*) and predictions by Radjai *et al.* (*28*). An earlier prediction by Radjai *et al.* (*28*) stated that the interparticle force network of static jammed materials consists of a subnetwork of strong interactions embedded in another subnetwork of weak interactions. Further investigations have shown that strong forces form linear filamentary chains that are stabilized by weak interactions (*15*). One can see in Fig. 1A that the largest cluster consists of polygons, and neighboring polygons are hinged by triangular structures. The formation of such closed loops, which resemble various types of polygons, for frictionless systems is quite unexpected, because frictional forces are required to stabilize arches. For this case, an arch is balanced by embedding weak clusters. This is in accord with Arevalo *et al.* (*15*). At the critical transition point (Fig. 1B), the yellow cluster contains most of the strong interactions, and, when most of the weak interactions are diluted in (Fig. 1C), the largest cluster contains mostly filamentary chains. Regardless of the value of *f*_t_, triangles seem to have the largest population, which is consistent with previous studies (*15*): The number of triangles only changes as a function of the coordination number that does not change when *f*_t_ is varied. One can see that these topological properties, which were mostly identified for static jammed matter, are rather universal, and they govern the structure of flowing matter as well.

### Exponents of percolation at $\dot{\gamma }\;\leq \;{\dot{\gamma }}_{c}$

**Fig. 2.**
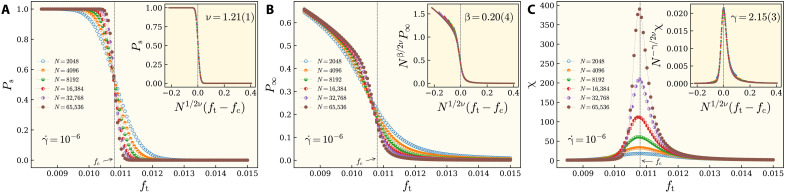
Exponents of the RP transition at vanishing rate. Finite-size scaling is performed at the vanishing limit of flow rate at $\dot{\gamma }=10^{-6}$, and the packing fraction is ϕ = 0.86. (**A**) The percolation probability *P*_s_ along either side of the system for various sizes is depicted. All curves cross at a common point at *f*_c_ signaling the critical nature of the system at the transition point. It is expected that, at the infinite system size, *P*_s_ becomes a step function. As the number of particles is increased, *P*_s_ approaches a step function. According to Eq. 1, by a rescaling according to *N*^1/2ν^(*f* − *f*_c_), all curves must collapse into a master function. We obtain an excellent collapse in the inset for ν = 1.21 ± 0.01. (**B**) The percolation strength *P*_∞_ is depicted for different sizes. A rescaling according to Eq. 2 results to a data collapse at the inset where β = 0.20 ± 0.04. (**C**) Mean cluster size, where the largest cluster is excluded, is depicted for various system sizes. Using Eq. 3, a scaling collapse is achieved for γ = 2.15 ± 0.03 at the inset. A comparison of these exponents with those of the central force RP (*18*) reveals that the universality class obeys the standard RP. Each data point is an average over an ensemble of at least 1.5 × 10^4^ configurations. Figures S1 to S6 present the details of finite-size scaling and data collapse for various $\dot{\gamma }$.

We now characterize the nature of the percolation transition of interparticle forces using scaling analysis of the divergence of correlation length, ξ ∼ ∣*f*_t_ − *f*_c_∣^−ν^; the scaling of the percolation strength, *P*_∞_ ∼ ∣*f*_t_ − *f*_c_∣^β^ as the order parameter; and the mean cluster size, χ ∼ ∣*f*_t_ − *f*_c_∣^−γ^, which acts as the susceptibility in the percolation transition. ν, β, and γ are three critical exponents characterizing the universality class of transition. These exponents can be computed by direct fitting of these functions to the corresponding data. However, in doing so, these exponents will be system size dependent. The universality class of the transition can only be determined, when the values of these exponents are unaffected by the system size. Finite-size scaling is a powerful tool for this purpose, which enables one to extrapolate the exponents corresponding to a system with infinite size. We perform systematic finite-size scaling analyses of the percolation probability *P*_s_, defined as the fraction of configurations that contain a spanning cluster along the *x* or *y* direction; the percolation strength *P*_∞_, defined as the probability that a site belongs to the spanning cluster; and susceptibility χ, defined as the mean cluster size excluding the largest cluster. We assume the following scaling laws

$$P_{s}=G_{1}\left[N^{\frac{1}{d\nu }}(f_{t}-f_{c})\right]$$ (1)

$$P_{\infty }=N^{\frac{-\beta }{d\nu }}G_{2}\left[N^{\frac{1}{d\nu }}(f_{t}-f_{c})\right]$$ (2)

$$\chi =N^{\frac{\gamma }{d\nu }}G_{3}\left[N^{\frac{1}{d\nu }}(f_{t}-f_{c})\right]$$ (3)

where G*_i_*,*i* = 1, 2, and 3 are scaling functions; *d* is the dimension of the space (*d* = 2 in our case); and *N* is the number of particles. These equations convey the fact that *P*_s_ approaches a step function as *N* → ∞, *P*_∞_ converges according to *N*^−β/*d*ν^, and χ diverges according to *N*^γ/*d*ν^ at *f*_t_ = *f*_c_. In Fig. 2 (A to C), we show *P*_s_, *P*_∞_, and χ, respectively, as a function of a varying force threshold *f*_t_ for various system sizes displayed by different colors. The flow rate is $\dot{\gamma }=10^{-6}$ for all these panels. One can see that *P*_s_ approaches a Heaviside function as *N* → ∞, and all curves corresponding to different system sizes cross at *f*_c_. This demonstrates that *P*_s_ becomes scale invariant at *f*_t_ = *f*_c_ signaling the critical nature of the underlying percolation transition. In Fig. 2A (inset), we obtain an excellent collapse of our data into a master curve after rescaling the horizontal axis according to Eq. 1 with *N*^1/*d*ν^(*f*_t_ − *f*_c_), where ν = 1.21 ± 0.01. The best collapse of *P*_∞_ according to Eq. 2 in Fig. 2B (inset) suggests β = 0.20 ± 0.04, and, similarly, we get γ = 2.15 ± 0.03 from an excellent collapse of our data according to Eq. 3 in Fig. 2C (inset).

A comparison of our ν and β exponents with those by the central force RP (*18*) unequivocally establishes that the universality class is the standard RP. To the best of our knowledge, this is the first verification of standard RP in an off-lattice molecular dynamics simulation. This paves the way to examine RP in other model glass formers and experiments. An ensuing important question is that does the flow rate acts as a relevant parameter in a sense that its variation could drive the universality of the system along a continuous line of classes? In a recent study, it was found that the spatial correlation between bonds in a lattice model for gelation acted as an irrelevant perturbation (*22*).

### Exponents of percolation at $\dot{\gamma }>{\dot{\gamma }}_{c}$

**Fig. 3.**
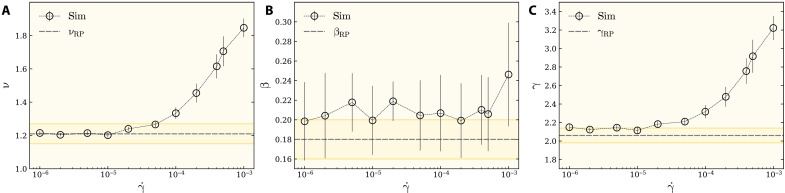
Exponents of the percolation transition at finite rates. We vary the flow rate and compute the exponents of percolation transition via finite size scaling for ν, β, and γ exponents in (**A**) to (**C**), respectively. The horizontal dashed line in (A) to (C) shows the RP exponents, and the shaded area is the corresponding error bar of the exponent. Most of the exponents for $\dot{\gamma }<10^{-5}$ more or less lie in the standard RP within the error bar. For $\dot{\gamma }>10^{-5}$, all exponents depart from RP. The packing fraction is ϕ = 0.86, and each data point is an average over an ensemble of at least 1.5 × 10^4^ configurations. In the Supplementary Materials, details of the estimation of error bars of exponents are given. Figures S1 to S6 depict finite-size scaling of the abovementioned critical exponents for various shear rates, $\dot{\gamma }$, which result in the true estimation of the critical exponents in the infinite size limit *N* → ∞.

To address whether the flow rate acts as a relevant parameter, we vary the flow rate in a range of three orders of magnitude from $\dot{\gamma }=10^{-6}$ to 10^−3^, and, for each flow rate, we perform a systematic finite-size scaling analysis similar to the aforementioned procedure, to compute critical exponents as a function of the flow rate. Owing to the very large system sizes simulated here, this is a very demanding task, which requires huge processing power and memory storage. We display ν, β, and γ as a function of the flow rate in Fig. 3 (A to C), respectively, which are estimated in the thermodynamic limit *N* → ∞. One can see that the flow rate manifests itself as a relevant perturbation and that gives rise to a spectrum of exponents. The horizontal dashed lines in Fig. 3 (A and B) display RP, and the gray bands show the corresponding uncertainty for RP exponents reported in the existing literature. Note that for $\dot{\gamma }\leq {\dot{\gamma }}_{c}≃10^{-5}$, all data points remain within the RP band. As a result, for small flow rates, the universality class of the percolation transition remains within RP, but a departure from RP happens at larger flow rates. However, the vanishing limit of the flow rate is firmly RP. The correlation exponent ν increases markedly for $\dot{\gamma }>{\dot{\gamma }}_{c}≃10^{-5}$. Similar observations have been reported for plastic events in sheared amorphous materials, in which, at low flow rates, plastic events are correlated, yet, at large rates, these events become uncorrelated and random (*29*, *30*).

### Force-force correlations

**Fig. 4.**
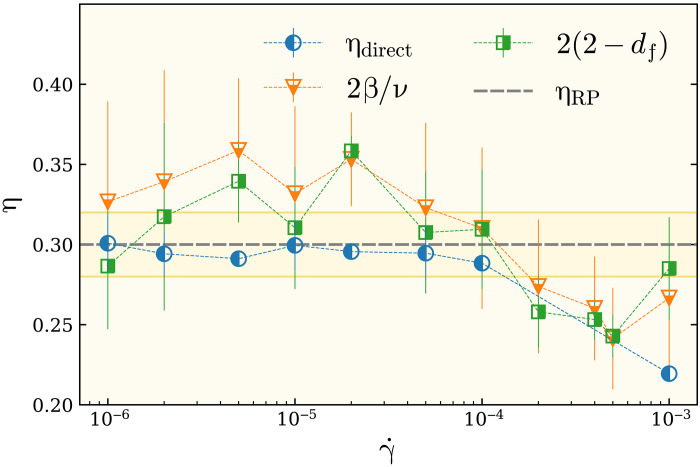
Exponent of force-force cluster correlation. η is the exponent of force-force cluster correlation calculated via the scaling of pair correlation function *g*(*r*) ∼ *r*^−(*d*−2+η_direct_)^ (circle) and derived from the hyperscaling relation η = 2β/ν (triangle) at *f*_t_ = *f*_c_ (see also fig. S7). The horizontal dashed line shows the RP exponent, and the shaded area is the corresponding error bar of the exponent estimated in previous works. η Does not display a systematic dependence in the wide range of flow rate $\dot{\gamma }$. It is shown that the nature of force-force cluster correlation is given by that of RP for all flow rates. The packing fraction is ϕ = 0.86, and each data point is an average over an ensemble of at least 1.5 × 10^4^ configurations.

Stress/force correlations have been found in both athermal jammed systems (*31*–*33*), inherent state of supercooled liquids and glasses (*12*, *34*–*39*), sheared granular media (*33*, *40*), and quiescent liquids (*38*). A typical stress correlator scales with the distance *r* as 1/*r^d^*, where *d* is the dimension of the space. It is now generally believed that the origin of stress correlation in amorphous materials is the mechanical equilibrium, Newton’s law of force and torque balance. This has been elaborated in recent field theoretic treatments of amorphous materials (*31*, *41*, *42*). However, for flowing amorphous material, in which the condition of mechanical equilibrium is broken, the nature of correlations remains elusive. Our percolation framework provides an explicit calculation of some sort of force-force correlations via the scaling of the pair-site correlation function. According to percolation theory (*8*), two particles separated by a distance *r* are likely to belong to the same cluster of force chains of strength *f* ≥ *f*_c_, by a probability proportional to *g*(*r*) ∼ *r*^−(*d*−2+η)^. In Fig. 4, we show η as a function of the flow rate (see fig. S7 for details of our computations for the correlation function and estimation of the exponent η in the infinite size limit *N* → ∞ for various $\dot{\gamma }$). η Does not show a systematic dependence in a wide range of flow rates. Moreover, independent of the flow rate, the correlation exponent is given by that of RP (complete results are presented in figs. S1 to S8).

### Theoretical framework

Our findings suggest that the flow rate $\dot{\gamma }$, as the only parameter in our problem, plays the role of a relevant operator that introduces long-range correlated disorder in our model and can alter the universality of the pure system at $\dot{\gamma }\to 0$. To elucidate our proposition, we draw upon the theoretical framework articulated by the Harris criterion (*43*). According to this criterion, short-range correlations with a falloff faster than *r*^−*d*^ are relevant if *d*ν − 2 < 0, where ν is the correlation length exponent of the pure model, i.e., that of the RP at $\dot{\gamma }\leq {\dot{\gamma }}_{c}$ in our system with ν ≃ 1.21 in two dimensions. Because we have *d*ν − 2 > 0, so the effective correlations induced by the flow rate in our system cannot be short range but essentially long range. For the induced long-range correlations of the power-law form C(*r*) ∼ *r*^−2*H*^ with 2*H* < *d*, the extended Harris criterion (*44*) then predicts that the correlations are relevant if *H*ν − 1 < 0. This gives the new correlation length exponent by the scaling relation ν*_H_* = 1/*H*. This relation has been extensively verified numerically in various studies in the past (*45*–*49*). In the context of self-affine surfaces (related to our discussion here after we make an analogy with our model for a realization of force profile), it is shown (*50*) that, for *H* < 0, the percolation is not critical even in the thermodynamic limit and the self-averaging breaks down. For long-range correlated force configurations with 0 ≤ *H* < 1 (*d* = 2), in contrast, the transition is critical, and the self-averaging is recovered, which is in agreement with our observation for various flow rates in this study. The correlation length exponent in the latter case is then shown to be given as follows in terms of the value of *H*

$$\nu_{H}=\left\{ \begin{array}{ccc} 1/H & \mathrm{if} & 0<H<1/\nu \\ \nu & \mathrm{if} & H\geq 1/\nu \end{array}\right.$$ (4)

**Fig. 5.**
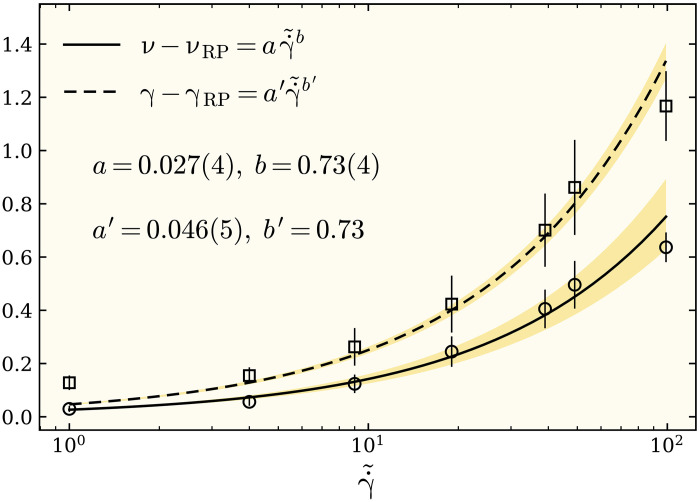
Power-law scaling of critical exponents. Above a crossover flow rate ${\dot{\gamma }}_{c}≃10^{-5}$, the critical exponents ν and γ increase algebraically with the reduced flow rate $\tilde{\dot{\gamma }}=(\dot{\gamma }-{\dot{\gamma }}_{c})/{\dot{\gamma }}_{c}$. Both exponents share the same scaling relation $∼{\tilde{\dot{\gamma }}}^{0.73}$ within the error bars. The shaded regions denote the SE in the fit exponent and the amplitudes over the baselines. *a* and *b* are obtained from the best power-law fit to our data for the correlation exponent ν, *b*′ = *b* = 0.73, and *a*′ is considered as the only fit parameter for $\dot{\gamma }$. The packing fraction is ϕ = 0.86, and each data point is an average over an ensemble of at least 1.5 × 10^4^ configurations.

Although this was originally shown for the random percolation model, we note that the same scenario is running here too. According to our results for the exponents presented in Fig. 3A, for small flow rates of roughly $\dot{\gamma }\leq 10^{-5}$, we find the same correlation exponent ν*_H_* ≃ 1.21 as for the standard RP, for which the decay of force-force correlations is governed by the value *H* ≥ 0.825 ± 0.01. In the limiting case $\dot{\gamma }\ll {\dot{\gamma }}_{c}$ (where *H* → 1), the correlations will be given by the marginal case proportional to ∝*r*^−*d*^ in *d* = 2. As the flow rate increases beyond the value ${\dot{\gamma }}_{c}≃10^{-5}$, the correlations induced by the flow rate become long range enough to change the correlation length exponent toward higher values. In particular, we find that, for $\dot{\gamma }≃10^{-4}$, the critical exponents are in agreement with the random percolation universality class with ν = 4/3, thus giving a proper suggestion *H* = 3/4 for the decay of the correlation function, which is in agreement with the previous results in (*51*). In the infinite flow rate limit $\dot{\gamma }\to \infty$, our theoretical arguments suggest that *H* → 0, i.e., logarithmic correlation functions appear, which are the characteristic feature of turbulence as a strongly fluctuating systems in two dimensions (*52*). The best fit to our data for the critical exponent ν provides the following relation with the flow rate, consistent with our above arguments

$$\nu -\nu_{\mathrm{RP}}=\left\{ \begin{array}{ccc} a{\tilde{\dot{\gamma }}}^{b}\; & \mathrm{if} & \dot{\gamma }>{\dot{\gamma }}_{c}\\ 0\; & \mathrm{if} & \dot{\gamma }\leq {\dot{\gamma }}_{c} \end{array}\right.$$ (5)

where $\tilde{\dot{\gamma }}=(\dot{\gamma }-{\dot{\gamma }}_{c})/{\dot{\gamma }}_{c}$, ν_RP_ = 1.21 denotes the exponent for the RP, *a* = 0.027(4), and *b* = 0.73(4) (Fig. 5). The crossover flow rate ${\dot{\gamma }}_{c}≃10^{-5}$ is roughly the point where the system crosses over from a rigid state to the rapid flow regime.

To further evaluate the geometric response of the force chain structure to the flow rate, we have also measured the fractal dimension *d*_f_ of the largest force cluster at *f* = *f*_c_ as a function of $\dot{\gamma }$. The fractal dimension can be estimated from the scaling relation *M* ∼ *N*^*d*_f_/2^, with *M* being the average number of nodes that belong to the percolating force cluster. Our results are presented in Fig. 4 in the form 2(2 − *d*_f_) to further test whether it fulfills the hyperscaling relation η = 2 + *d* − 2*d*_f_ (see fig. S8 for more details). As shown in Fig. 4, this scaling relation seems to be valid within the error bars, with an additional observation that the exponents in Fig. 4 show a weak dependence over the whole range of considered $\dot{\gamma }$. To assess the validity of this observation, we take advantage of the scaling relation η = 2 − γ/ν, according to which, in order for η to be independent of $\dot{\gamma }$, the dependence of γ and ν must be canceled out. According to Eq. 5, this requires $\gamma -\gamma_{\mathrm{RP}}=a^{\prime }{\tilde{\dot{\gamma }}}^{b\prime }$ with *b*′ = *b* = 0.73. Figure 5 shows our best fit $a^{\prime }{\tilde{\dot{\gamma }}}^{0.73}$ to the data for γ − γ_RP_ as a function of $\tilde{\dot{\gamma }}$ for $\dot{\gamma }>{\dot{\gamma }}_{c}$, which gives *a*′ = 0.046(5). This gives back η = 2 − *a*′/*a* ≃ 0.30, which is in perfect agreement with our observation shown in Fig. 4. It must basically be possible to check similar behavior for the exponent β, but, because our data presented in Fig. 3B bear rather large error bars, our test was not conclusive enough to be shown in Fig. 5.

## DISCUSSION

The interplay between network topology and macroscopic properties in network glass formers (*53*), and more specifically in chalcogenide network glasses (*54*, *55*), has been well addressed in the context of RP (*18*). Attempts have been made to reconcile RP with isotropic jamming (*21*), yet such a connection has been evasive. The reconciliation of RP with jammed granular media is crucial because such a reconciliation will facilitate a unified framework for granular media and glasses, as suggested by long-range stress correlations in both systems.

Here, we investigated the percolation of interparticle forces between particles in a dense regime in which all degrees of freedom of particles are over-constrained (*56*). We unequivocally established that the connectivity of the interparticle force network undergoes a second-order phase transition, whose scaling properties, for a wide range of flow rates, are given by those of the RP universality class. This demonstrates that the emergence of shear elasticity originates from the internal organization of particles in the form of chains of interparticle forces. Thus, the emergence of shear elasticity is a manifestation of mechanical self-organization. Recent work supports our view; e.g., Tong *et al.* (*12*) investigated stress correlation in a model glass at finite temperature with giant anharmonic fluctuations and demonstrated that the stress correlation emerges as a result of long-range interparticle force correlation manifested as a distinct correlated percolation universality class, a hint to mechanical self-organization.

A crucial step toward a better understanding of force/stress anomalies requires lifting the constraint of the mechanical balance. This is very important for parent liquid states and flowing granular media that are not quenched into a local energy minimum. As pointed out by Lemaître (*38*), this is very challenging because, in the absence of the mechanical equilibrium, the structure of correlations becomes very complex. Our work is a step forward in this direction. It provides a previously unknown link between force correlation in flowing granular media, RP, and a potential relationship to mechanical self-organization. The ingredients giving rise to RP in sheared frictionless spheres, namely, (i) over-constraining and (ii) slow deformation, are important and may motivate future attempts for field theoretic treatments (*31*, *41*) of amorphous flowing material.

Our results cover a typical athermal system undergoing a shear-driven jamming transition in the dense phase. An important question is whether and to what extent these results apply to thermal glasses. The RP has been originally devised to address the glass transition in thermal glasses, yet it has not been confirmed in any molecular simulation. Determining whether a slow deformation is a necessary factor for recovering RP in thermal glasses will be an exciting research pursuit for future numerical investigations. We hope our work will inspire further studies on thermal glasses using molecular dynamics simulations.

A recent study on jamming by compression shows that the compression rate is a relevant parameter for the jamming transition (*57*). Moreover, the authors find that isotropically compression-driven jamming is in the same universality class as that of shear-driven jamming. Another study shows that, whereas jamming by compression is markedly history dependent and, as a result, gives rise to a range of critical densities, shear-driven jamming is not history dependent (*58*). There is a chance that the controversy in percolation in isotropically compressed packings is related to these issues and the history of the system plays the role of a relevant parameter giving rise to a spectrum of critical exponents. Our results should motivate future works to resolve this controversy.

## METHODS

Our system consists of frictionless bidisperse disks in two-dimensional space. The interactions between disks are governed by short-range linear repulsive and dissipative forces. These interactions can be expressed as **F***_ij_* = *K_n_*ξ*_ij_***r***_ij_*/*r_ij_* − *M*_eff_γ*_n_***v***_n_*. Two particles *i* and *j* of radii *R_i_* and *R_j_* at positions **r***_i_* and **r***_j_* interact when the mutual compression of particles ξ*_ij_* = *R_i_* + *R_j_* − *r_ij_* > 0. In this equation, **r***_ij_* = **r***_i_* − **r***_j_*, *K_n_* is the elastic constant for a normal contact, and γ*_n_* is the viscoelastic damping constant for a normal contact. **v***_n_* is the normal component of the relative velocity of the two particles, and *M*_eff_ = *m*_1_*m*_2_/(*m*_1_ + *m*_2_). The ξ*_ij_* is called the mutual compression of two particles. We use LAMMPS software (*27*) for simulating shear-driven granular systems. The Verlet method (*59*), which serves as the default integrator in LAMMPS, is used to numerically integrate the equations of motion for particles. This algorithm calculates the new positions and velocities of particles using their current values and the forces acting on them by using a two-step process that accounts for both position and velocity updates. The details of the algorithm are incorporated in section S3. Lees-Edwards boundary conditions (*59*) are applied to create a uniform overall flow rate $\dot{\gamma }$ along the *x* direction. To emulate these boundary conditions in LAMMPS, each particle is given an initial velocity according to $v_{i}(t=0)=\dot{\gamma }y_{i}\hat{i}$ at time *t* = 0, where *y_i_* is the *y* position of particle *i*. To maintain the velocity profile, we use the command “fix deform” with “remap v” option.

To prevent crystallization, we use a 1 : 1 binary mixture of particles with particle radii *R*_0_ = 0.5 and *R*_1_ = 0.7. For seeking simplicity, we set the mass of each particle equal to its area, *m* = π*R*^2^. By considering *K_n_* = 1 and γ*_n_* = 1, we measure time in our simulations using the units of τ_0_ ≡ γ*_n_*$d_{0}^{2}$/*K_n_* = 1, where *d*_0_ represents the diameter of the small particles. The shear rate can then be nondimensionalized with $\dot{\gamma }\times \tau_{0}$, which is important for comparison with other simulations and experiments. The packing fraction ϕ = 0.86 > ϕ*_J_* is considered. To check the robustness of the results against changing the packing fraction and the repulsive force amplitude, we simulate systems with ϕ = 0.865, *K_n_* = 1, and ϕ = 0.86, *K_n_* = 1.5, as shown in figs. S9 to S13. Notably, we find that these changes do not significantly affect the reported original exponents, as they fall within the same range of error bars (see fig. S13).

The number of particles is *N* = 2048, 4096, 8192, 16,384, 32,768, and 65,536. This vast range of system sizes facilitates a systematic finite-size scaling, by which we calculate all the scaling properties of networks at the thermodynamic limit *N* → ∞. Furthermore, various flow rates $\dot{\gamma }=$ 10^−6^, 2 × 10^−6^, 5 × 10^−6^, 10^−5^, 2 × 10^−5^, 5 × 10^−5^, 10^−4^, 2 × 10^−4^, 4 × 10^−4^, 5 × 10^−4^, and 10^−3^ are considered for each system size. All reported quantities, such as *P*_s_, *P*_∞_, and χ are computed by averaging over 10 independent simulations (realizations) each of which consists of around 1.5 × 10^3^ uncorrelated configurations separated by a strain difference equal to the unit of the length after the system has reached a steady state.

## Supplementary Materials

### This PDF file includes:

Sections S1 to S4 Figs. S1 to S13
