# Supplementary material for: Emergence of rigidity percolation in flowing granular systems
Source: Sci Adv. 2023 Sep 1;9(35):eadh5586. doi: 10.1126/sciadv.adh5586 (PMC12488052; doi:10.1126/sciadv.adh5586)
Supplement: Supplementary file 1 — Sections S1 to S4 Figs. S1 to S13 [file sciadv.adh5586_sm.pdf]

Science Advances
AAAS

Supplementary Materials for

Emergence of rigidity percolation in flowing granular systems

Hor Dashti *et al.*

Corresponding author: Hor Dashti, e.dashti@uq.edu.au; Abbas Ali Saberi, ab.saberi@ut.ac.ir; Jürgen Kurths, kurths@pik-potsdam.de

Sci. Adv. **9**, eadh5586 (2023)
DOI: 10.1126/sciadv.adh5586

This PDF file includes:

Sections S1 to S4
Figs. S1 to S13

This Supplementary Information presents the results of our extensive computations conducted for various system sizes (N) and flow rates ($\dot{\gamma}$) . From these computations, we have determined the critical exponents discussed in the main text for a packing fraction of $\phi = 0.86$. In Section S2, we examine other parameters to ensure the reliability of our findings. Section S3 outlines the integration method used for the equations of motion. Lastly, in Section S4, we provide further elaboration on equations (4) and (5) in the main body of the text.

Section S1. STATISTICS FOR $\phi = 0.86$

Percolation probability: We calculate the percolation probability P_s in terms of threshold force f_t for each flow rate $\dot{\gamma}$ (see all main panels in Fig. S1). One can fit P_s to the function $(1 - \text{erf}(f_t - F_N)/\Delta_N)/2$ to obtain the effective threshold force F_N and the width of the percolation probability Δ_N. The critical forces are estimated in the thermody-namic limit by extrapolation the F_N to the infinite system size — i.e., $f_c(\dot{\gamma}) = F_{N\to\infty}(\dot{\gamma})$, which are shown in Fig. S2A. Using scaling relation $\Delta_N \sim N^{-1/2\nu}$ (Fig. S2B), we obtain the exponents $\nu(\dot{\gamma})$. To estimate the error bar of scaling exponents, the covariance matrix of the scaling function ($y = bx^a$) that has two parameters, the coefficient b and exponent a are computed. The square root of diagonal elements of a covariance matrix gives the uncertainty of fitting function parameters. It is worth noting that the error bars of reported scaling exponents (ν, β, γ, η, d_f) in this draft are obtained by calculating the covariance matrix.

Percolation strength: The strength (weight) of the percolating cluster, P_∞, is the probability that a site belongs to an infinite cluster. In Fig. S3, P_∞ in terms of f_t are plotted for various system sizes and flow rates. The β exponents are obtained from relation $P_\infty(f_t = f_c) \sim N^{-\beta/2\nu}$ (see Fig. S4) using $f_c(\dot{\gamma})$ and $\nu(\dot{\gamma})$ which have been already derived above.

Mean cluster size: The mean cluster size is defined as $\chi = \sum_s s^2 n_s / \sum_s s n_s$, where s is the cluster size, n_s denotes the cluster number (i.e., the number of clusters with size s), and summation excludes the giant cluster. In Fig. S5, we show χ as a function of f_t for various system sizes and flow rates. Given $\nu(\dot{\gamma})$, the exponents $\gamma(\dot{\gamma})$ are calculated using $\chi_{max} = \chi(f_t = f_c) \sim N^{\gamma/2\nu}$ (see Fig. S6).

Pair connectivity (correlation function): The correlation function is the probability

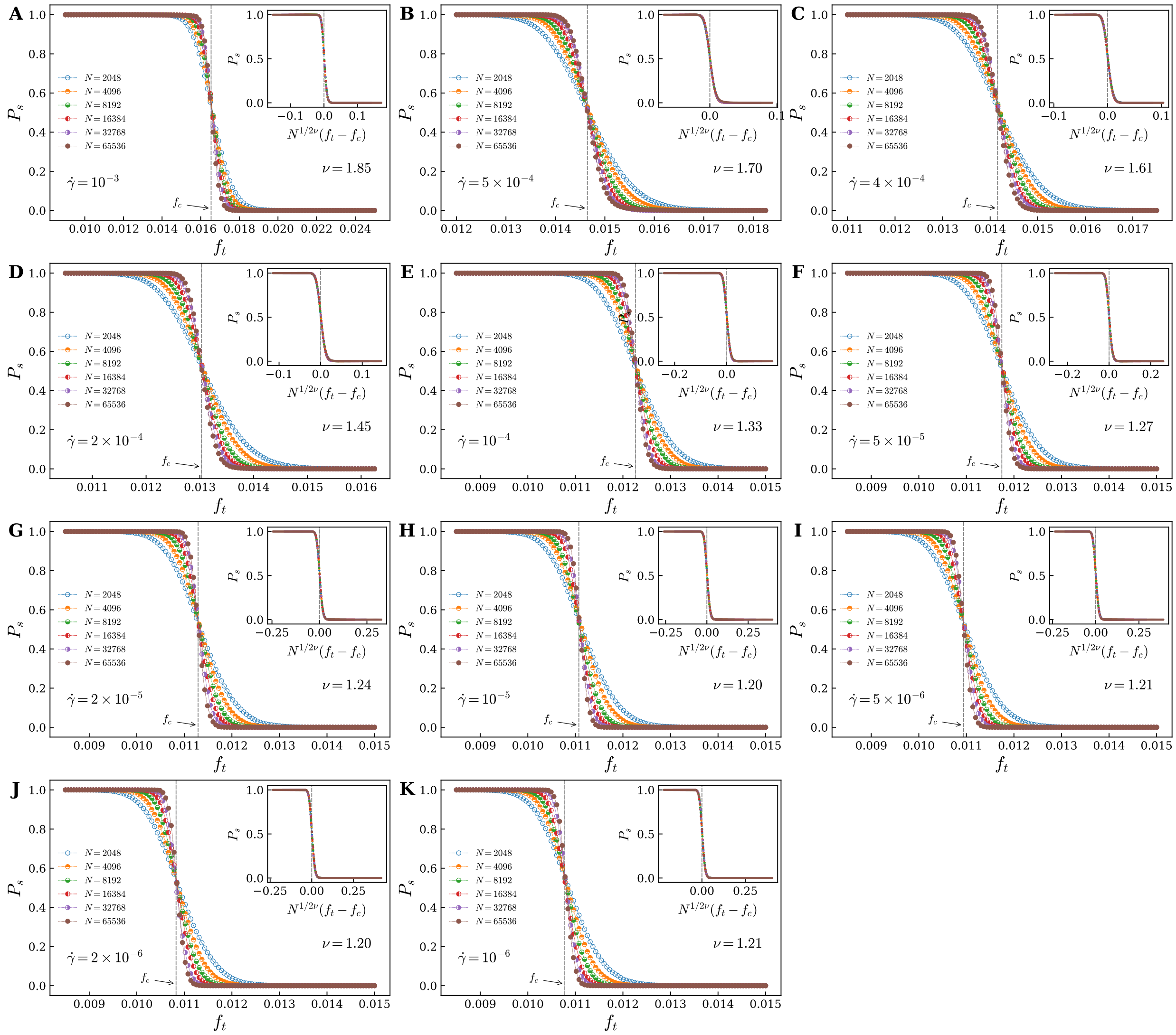

Fig S1. **Percolation probability.** Each panel corresponds to a flow rate showing the percolation probability P_s in terms of threshold force f_t for various system sizes N. The insets show the rescaled curves according to equation $P_s = \mathcal{G}_1(N^{\frac{1}{2\nu}}(f_t - f_c))$. Furthermore, the exponent ν is reported in each panel. The packing fraction is $\phi = 0.86$, and each data point is an average over an ensemble of at least 1.5×10^4 configurations.

that a site at position r from an occupied site belongs to the same cluster which follows the relation $g(r) \sim r^{-\eta}$ (Fig. S7).

Fractal dimension of infinite cluster: The mass (number of nodes) of infinite cluster at f_c follows the scaling relation $M \sim N^{d_f/2}$ which is shown in Fig. S8.

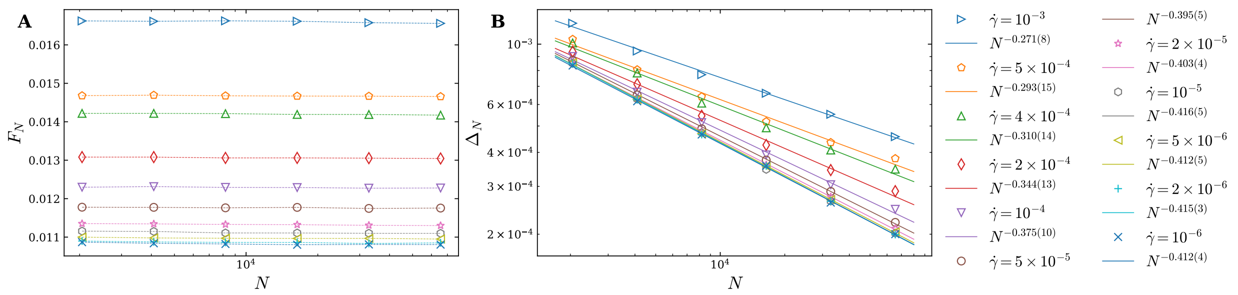

Fig S2. Panel-A and -B show the effective threshold force F_N and width of the percolation probability Δ_N, respectively, for each $\dot{\gamma}$ in terms of system size N. The error bar of each data point is less than symbol size. The critical force for each flow rate can be calculated using the relation $f_c(\dot{\gamma}) = F_{N\to\infty}(\dot{\gamma})$. Furthermore, we can see the scaling behavior of Δ_N versus N in panel-B. The exponents ν are derived by calculating the slope of each Δ_N curve in a log-log plot, which gives $1/2\nu$. The error of each curve's slope is represented in the legend of panel-B.

Section S2. STATISTICS FOR OTHER VALUES OF MODEL'S PARAMETERS

Previously, our model had parameters $\phi = 0.86$ and $K_n = 1$. Here, we present the dependence of critical exponents on different shear rates, in relation to other packing fraction values ϕ and elastic constant K_n.

Changing the packing fraction and the repulsive force amplitude: The percolation probability and mean cluster size in terms of threshold force for various system sizes and different model's parameters, ($\phi = 0.865$, $K_n = 1$) and ($\phi = 0.86$, $K_n = 1.5$), are shown in Figs. S9-S10, and S11-S12, respectively. Each data point represents an average over an ensemble of at least 3000 configurations. The shear-dependent exponents for these model parameters are depicted in Fig. S13. Notably, these exponents are approximately the same as those derived from the default parameters ($\phi = 0.86$, $K_n = 1$). However, due to the insufficient number of configurations, conclusive results for the shear-dependent exponent β could not be obtained, and therefore, they are not reported here. Nevertheless, the two other exponents, ν and γ, have been calculated and are shown in Fig. S13.

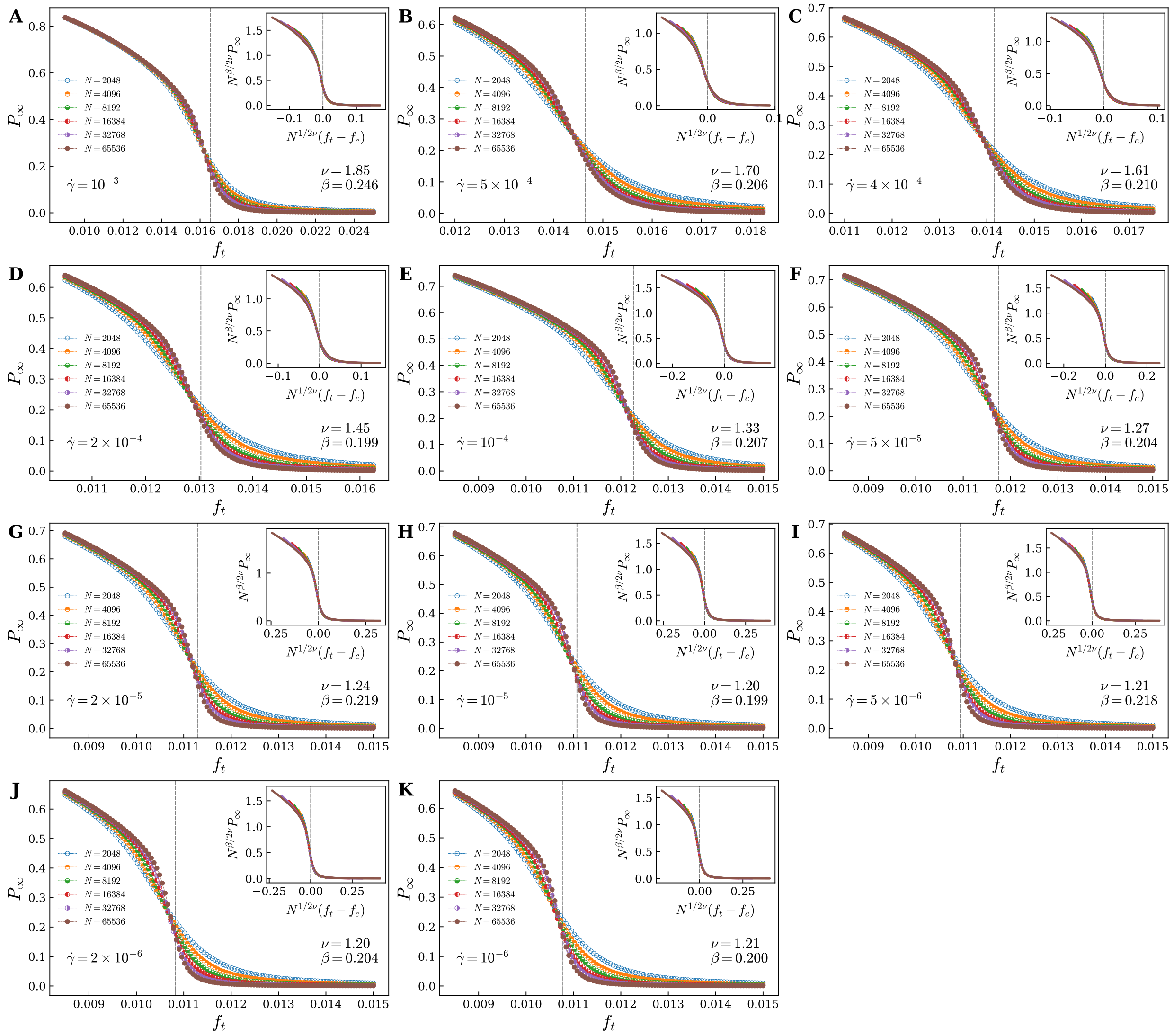

Fig S3. **Percolation strength.** In each panel relating to a flow rate $\dot{\gamma}$, the percolation strength $P_\infty(f_t)$ is plotted in terms of f_t for various system sizes N. The insets show the rescaled curves according to equation $P_\infty = N^{\frac{-\beta}{2\nu}}\mathcal{G}_2(N^{\frac{1}{2\nu}}(f_t - f_c))$. Moreover, the exponents β and ν corresponding to a flow rate are reported in each panel. The packing fraction is $\phi = 0.86$, and each data point is an average over an ensemble of at least 1.5×10^4configurations.

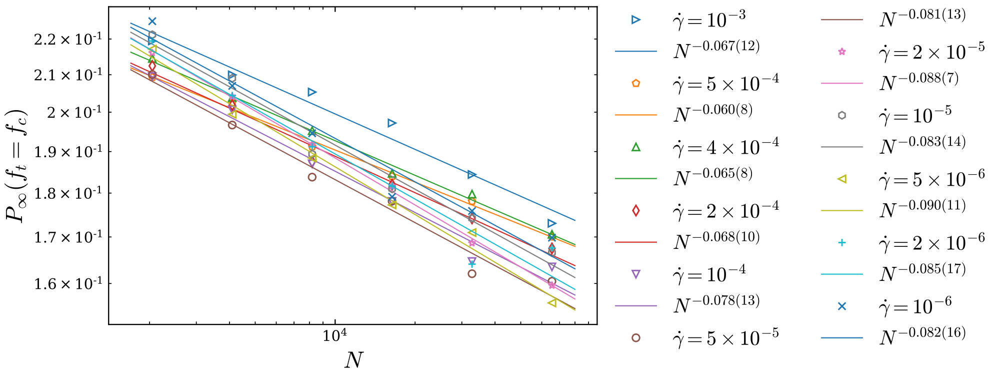

Fig S4. We vary the flow rate and calculate the percolation strength at critical force in terms of system size N. The slope of each curve in a log-log plot reads $-\beta/2\nu$. The slope values and their error bars are reported in the legend. Using $\nu(\dot\gamma)$ derived from Fig. S2B, we calculate the $\beta(\dot\gamma)$ exponents, which are reported in Fig. 3B of main text.

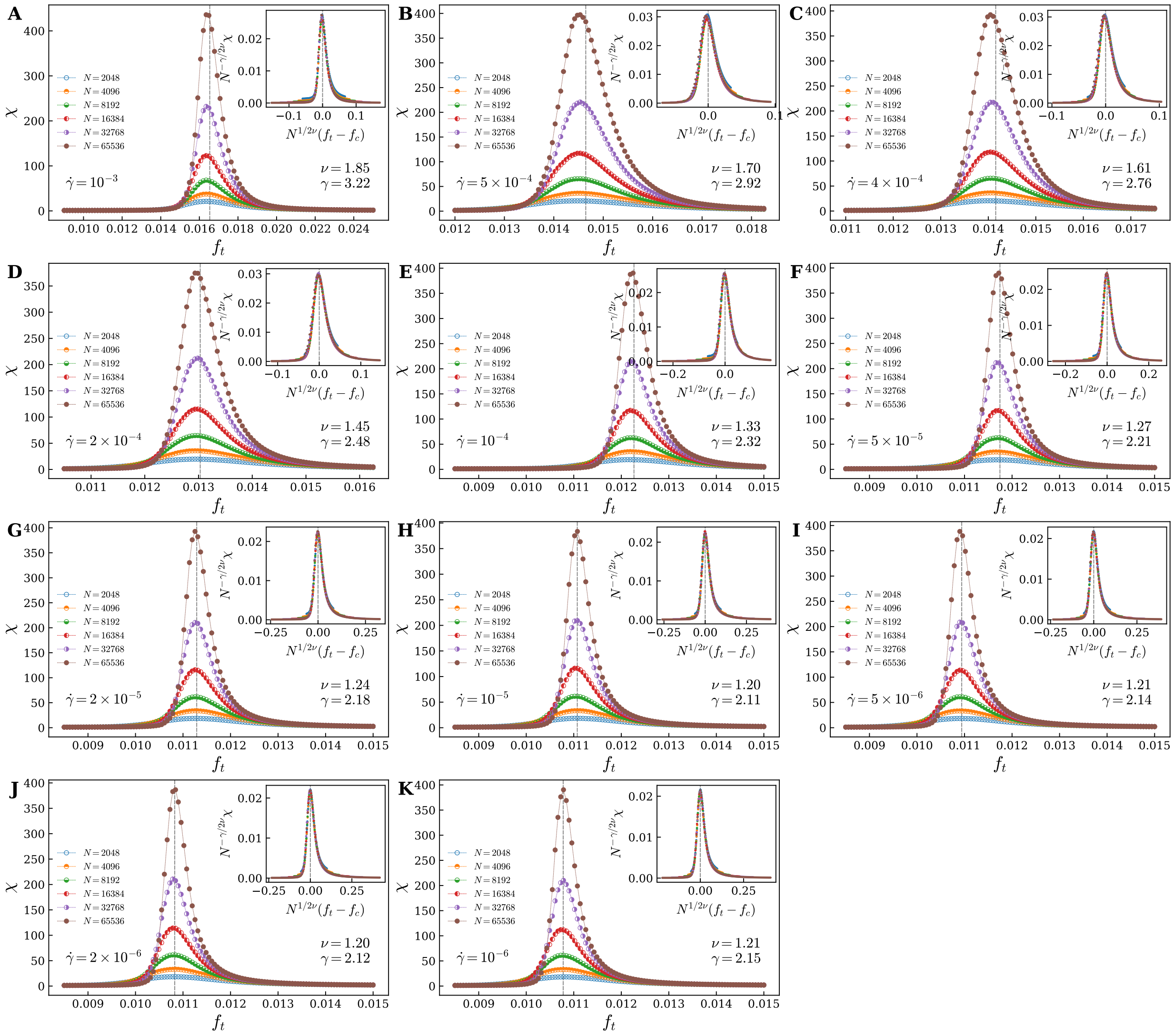

Fig S5. **Mean cluster size (susceptibility)** In each panel relating to a flow rate $\dot{\gamma}$, the susceptibility χ is plotted in terms of f_t for various system sizes N. The insets show the rescaled curves according to equation $\chi = N^{\frac{\gamma}{2\nu}}\mathcal{G}_3(N^{\frac{1}{2\nu}}(f_t - f_c))$. Furthermore, the exponents γ and ν corresponding to a flow rate are reported in each panel. The packing fraction is $\phi = 0.86$, and each data point is an average over an ensemble of at least 1.5×10^4 configurations.

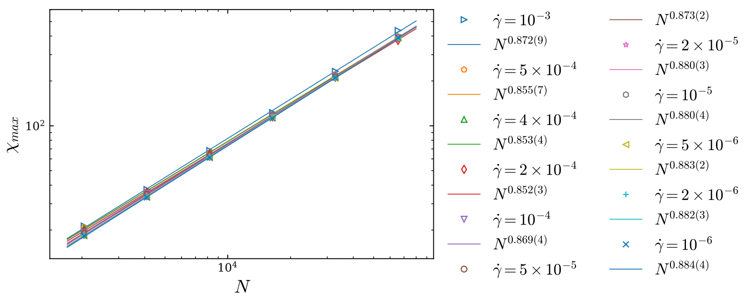

Fig S6. The mean cluster size at critical force in terms of system size N for various flow rates is shown. The slope of curves in a log-log plot read the exponents $\gamma/2\nu$. Given exponents $\nu(\dot\gamma)$ from Fig. S2, we calculate the exponents $\gamma(\dot\gamma)$, which are reported in Fig. 3C of main text.

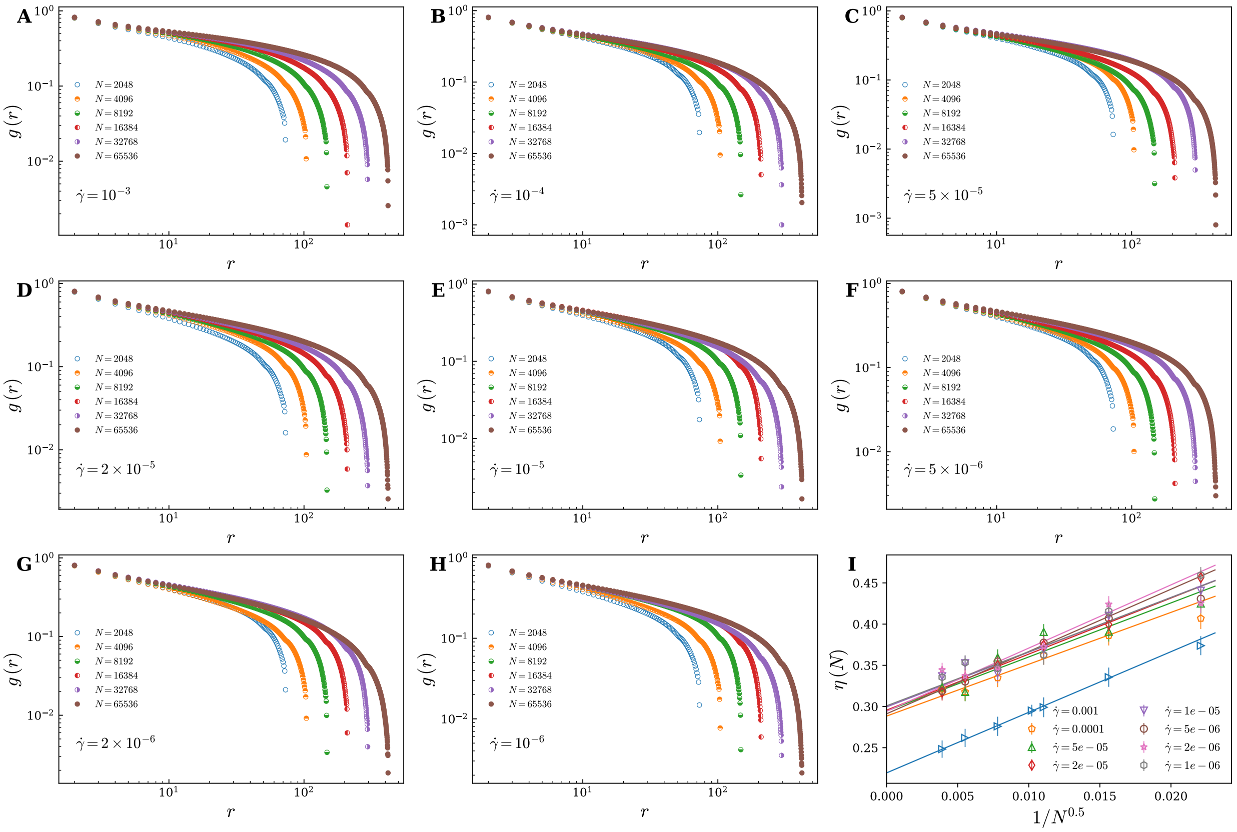

Fig S7. **Correlation function.** Panel-A to -H represent the correlation function $g(r)$ in terms of distance r for various system sizes N. Each curve satisfies the relation $g(r, N) \sim r^{-\eta(N)}$ for $r \ll r_{\text{cut-off}}$. So, the slope of each curve in log-log scale gives the exponent $\eta(N)$ (the anomalous dimension). In panel-I, the derived exponents $\eta(N)$ versus $1/N^{0.5}$ are plotted. The interception of each curve with y-axis reads the $\eta(N \to \infty)$. In Fig. 4 of main text, the exponent $\eta_{\text{direct}} = \eta(N \to \infty)$ is reported in terms of $\dot\gamma$.

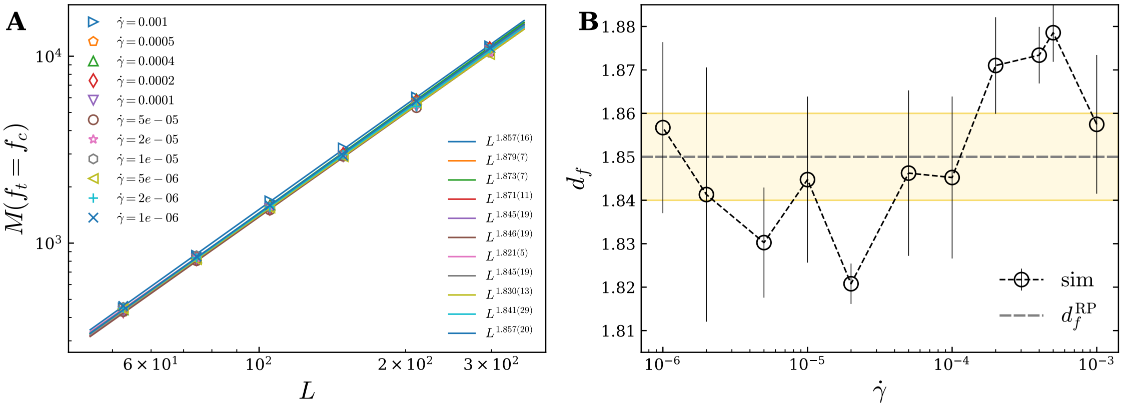

Fig S8. **Fractal dimension of infinite cluster.** The relation between the mass of infinite cluster M at $f_t = f_c$ versus L for various flow rates is shown in panel-A, which reads $M \sim L^{d_f} \sim N^{d_f/2}$. The fractal dimension $d_f(\dot\gamma)$ is plotted in panel-B. The horizontal dashed line shows the RP exponent and the shaded area is the corresponding error bar of the exponent.

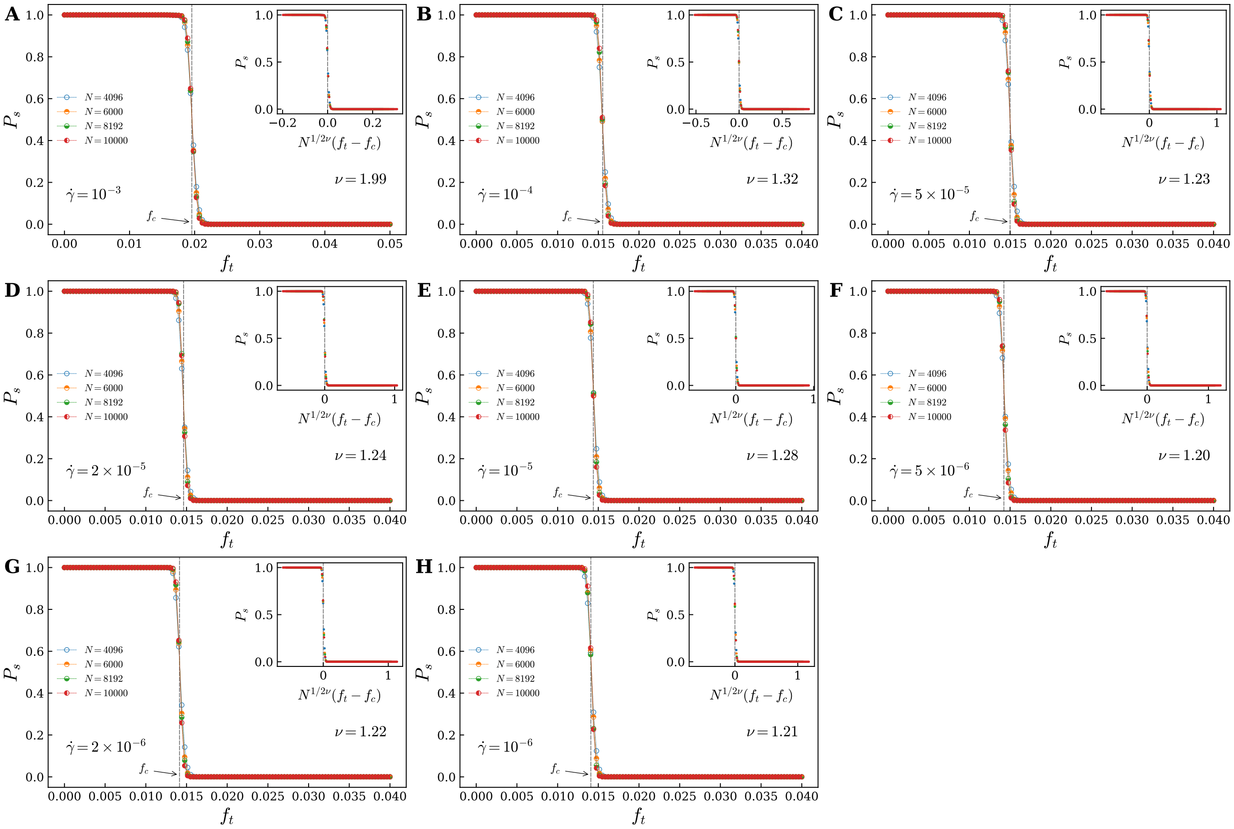

Fig S9. **Percolation probability.** P_s in terms of threshold force f_t for various system sizes N. The packing fraction $\phi = 0.865$ and elastic coefficient $K_n = 1$ are considered.

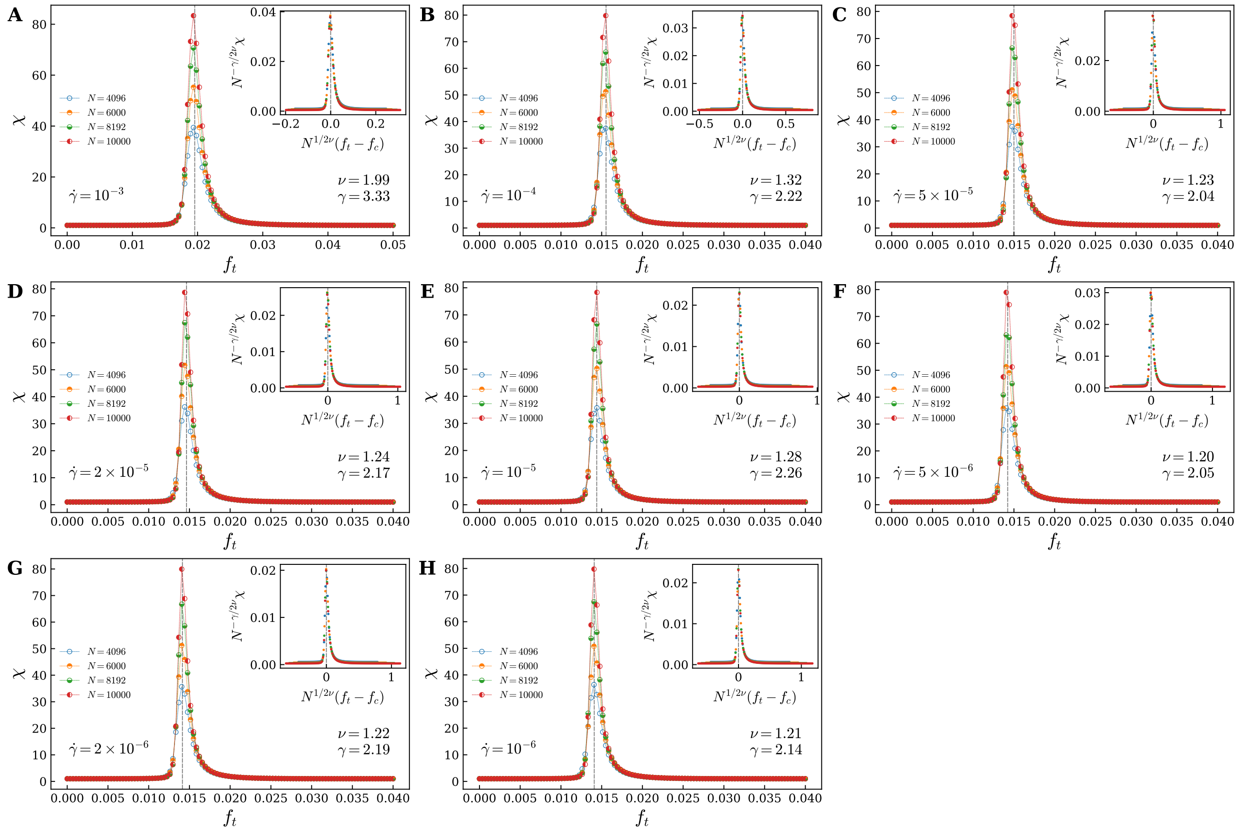

Fig S10. **Mean cluster size.** χ in terms of threshold force f_t for various system sizes N. The packing fraction $\phi = 0.865$ and elastic coefficient $K_n = 1$ are considered.

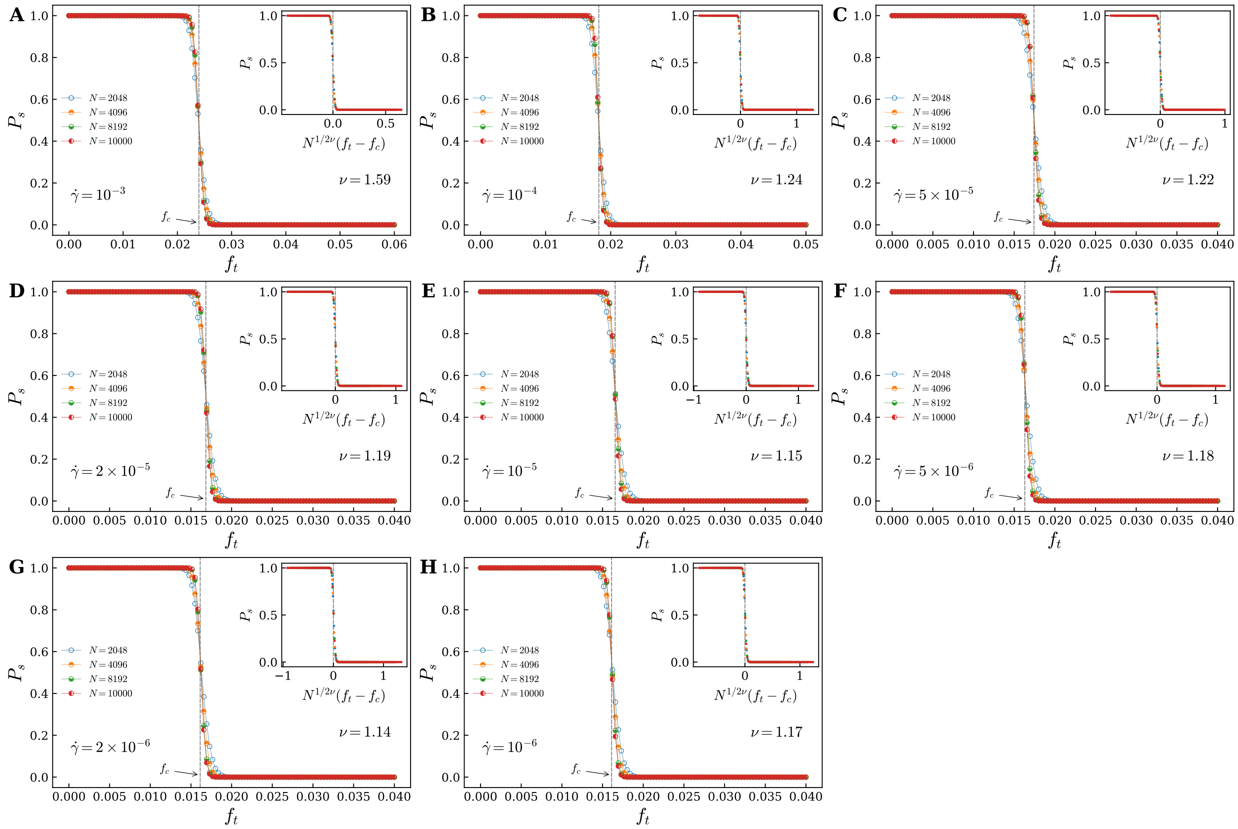

Fig S11. **Percolation probability.** P_s in terms of threshold force f_t for various system sizes N. The packing fraction $\phi = 0.86$ and elastic coefficient $K_n = 1.5$ are considered.

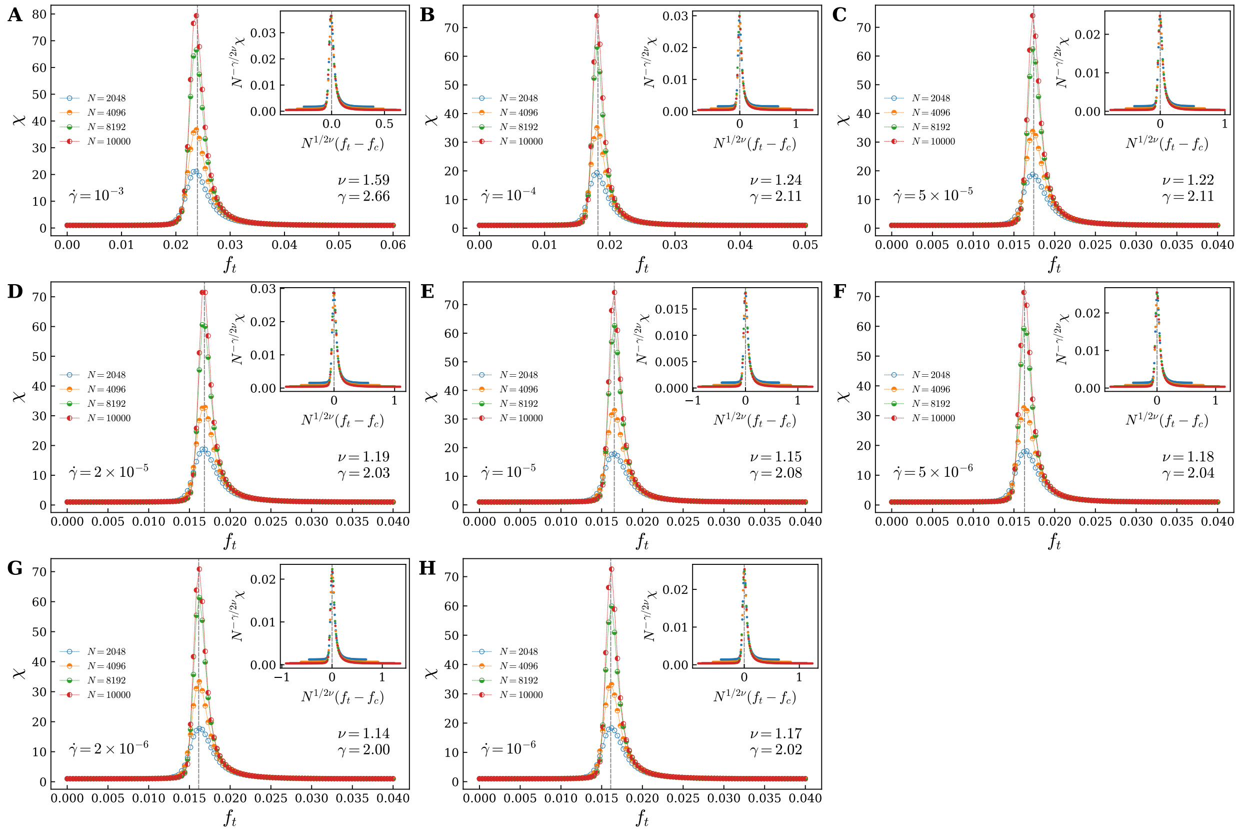

Fig S12. **Mean cluster size.** χ in terms of threshold force f_t for various system sizes N. The packing fraction $\phi = 0.86$ and elastic coefficient $K_n = 1.5$ are considered.

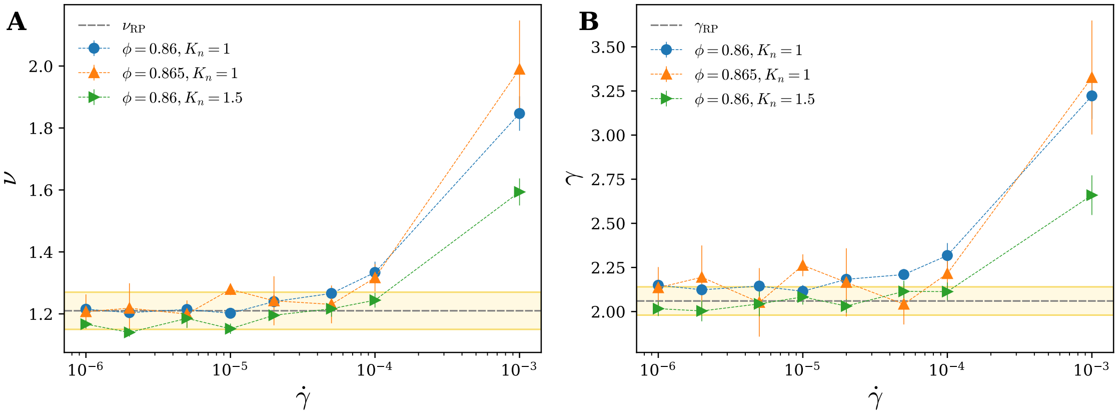

Fig S13. **Dependency of exponents to model's parameters.** The exponents ν and γ are plotted in terms of $\dot{\gamma}$ in panel-A and -B, respectively. By changing the model's parameters, the shear-dependent exponents exhibit similarity within the error bars to the exponents derived from the default parameters ($\phi = 0.86, K_n = 1$).

Section S3. INTEGRATING THE EQUATIONS OF MOTION

The Verlet integrator, known for its second-order accuracy, is used in LAMMPS to numerically integrate the equations of motion for particles. The pseudocode below illustrates how the Verlet method is implemented in LAMMPS (in the `src/verlet.cpp` file). In each time step, the new position and velocity of particle i are updated using the following relations:

$$\text{Update velocity by a half-step: } \mathbf{v}_i \leftarrow \mathbf{v}_i + \tfrac{1}{2}\delta t\, \mathbf{F}_i/m_i \tag{S1}$$

$$\text{Update position: } \mathbf{r}_i \leftarrow \mathbf{r}_i + \delta t\, \mathbf{v}_i \tag{S2}$$

$$\text{Apply the boundary conditions and box deforming} \tag{S3}$$

$$\text{Compute the force } \mathbf{F}_i \tag{S4}$$

$$\text{Update velocity by a half-step: } \mathbf{v}_i \leftarrow \mathbf{v}_i + \tfrac{1}{2}\delta t\, \mathbf{F}_i/m_i \tag{S5}$$

The force acting on particle i is denoted as $\mathbf{F}_i = \sum_j \mathbf{F}_{ij}$, where $\mathbf{F}_{ij}$ represents the interparticle interactions containing repulsive $(K_n \xi_{ij} \mathbf{r}_{ij}/r_{ij})$ and viscoelastic damping forces $(-M_{\text{eff}} \gamma_n \mathbf{v}_n)$;

$$\mathbf{F}_{ij} = K_n \xi_{ij} \mathbf{r}_{ij}/r_{ij} - M_{\text{eff}} \gamma_n \mathbf{v}_n$$

Two particles i and j of radii R_i and R_j at positions $\mathbf{r}_i$ and $\mathbf{r}_j$ interact when the mutual compression of particles $\xi_{ij} = R_i + R_j - r_{ij} > 0$, where $\mathbf{r}_{ij} = \mathbf{r}_i - \mathbf{r}_j$. K_n is the elastic constant for a normal contact, and γ_n is the viscoelastic damping constant for a normal contact. $\mathbf{v}_n$ is the normal component of the relative velocity of the two particles, and $M_{\text{eff}} = m_1 m_2/(m_1 + m_2)$.

Section S4. ADDITIONAL INFORMATION REGARDING THE THEORETICAL FRAME-WORK

In this section, let us briefly explain the main motivation behind the theoretical framework presented in the main text for understanding our numerical findings.

The original Harris criterion states that the presence of a *weak* disorder in a pure system can change the critical behavior of the system, depending on the value of the specific heat exponent $\alpha = 2 - d\nu$. If $\alpha > 0$, the disorder is relevant, meaning that it affects the critical behavior and leads to a new universality class. If $\alpha < 0$, the disorder is irrelevant, and the critical behavior remains the same as in the pure system. ν is the correlation-length

exponent for the pure system without disorder, which, in our case ($d = 2$), is approximately $\nu \simeq 1.21$, the same as that of rigidity percolation.

By placing these values ($\nu = 1.21$ and $d = 2$) in the hyperscaling relation it gives negative specific heat exponent $\alpha = 2 - d\nu = 2(1 - 1.21) < 0$. This means that a weak impurity is not able to change the universality class of our particular system, while our observation is that the critical features of our system are changed by introducing the flow rate $\dot{\gamma}$ in the system. Therefore, we propose a theoretical framework for understanding the change in the universality class of our system based on considering the flow rate $\dot{\gamma}$ *effectively* as an impurity in the system that induces extra correlations. In this setting, the system with $\dot{\gamma} \to 0$ denotes the pure system and the introduction of nonzero $\dot{\gamma}$ is *effectively* considered as the addition of an impurity to a pure system. Now, according to the introduction above, since in our system, we always have $\alpha < 0$, the induced correlations by the effective impurity which is basically responsible for the RG flow of the system to the new universality class can not be short-range/weak but essentially long-range. The effective long-range correlations induced by the impurity (i.e., the flow rate) are shown by the power-law decaying form $C(r) \sim r^{-2H}$, where $2H < d$ ensures its long-range extension and it implicitly assigns a corresponding H value to each value of $\dot{\gamma}$. This correspondence is further elucidated by Equations (4) and (5) in the main text. So, with $C(r)$ in the main text, we mean the range of the induced effective correlations by the flow rate, which should be distinguished from the force-force correlation function described by $g(r) \sim r^{-(d-2+\eta)}$.

In order to investigate the relevance of the long-range correlated disorder on the RG flow and the critical behavior of the model, one needs an extended version of the Harris criterion which goes beyond the original case introduced for the effect of weak disorder. The extended Harris criterion predicts that the correlations are relevant if $H\nu - 1 < 0$. Again, ν is the correlation-length exponent for the pure system without the disorder (or subject to irrelevant short-range disorder corresponding to $2H \gtrsim d = 2$) which is that of the rigidity percolation $\nu \simeq 1.21$.

The extended Harris criterion thus predicts that our model, for the range $H \geq 1/\nu \simeq 1/1.21 = 0.825 \pm 0.01$ corresponding to flow rates of $\dot{\gamma} \lesssim 10^{-5}$, has irrelevant contribution in the critical behavior of the system with an intact correlation length exponent $\nu_H = \nu \simeq 1.21$.

This further suggests that in the limit $\dot{\gamma} \to 0$ which corresponds to $H \to 1$, the correlation length exponent ν_H is given by the one for the pure system, i.e., $\nu_H \simeq 1.21$, which is also

supported by our data shown in Fig. 3 of the main text. Loosely speaking, We find that for $\dot{\gamma} \lesssim 10^{-5}$ (or equivalently $H \geq 0.825 \pm 0.01$) the flow rate has an irrelevant contribution to the critical behavior of the system while for $\dot{\gamma} > 10^{-5}$ (or equivalently $0 < H < 0.825 \pm 0.01$), it is relevant by driving the system away from its original critical behavior and induces new critical points described with genuine critical exponents.
